# Insecticide Resistance Alters Oviposition Preference in *Drosophila melanogaster*

**DOI:** 10.1002/ece3.73067

**Published:** 2026-02-09

**Authors:** A. Nogueira Alves, F. Martelli, Y. T. Yang, N. Wedell

**Affiliations:** ^1^ School of BioSciences University of Melbourne Melbourne Victoria Australia

**Keywords:** behaviour, *Cyp6g1*, DDT, imidacloprid, insecticides, insects, Spinosad

## Abstract

Environmental pressures, particularly those driven by anthropogenic activity, can induce rapid behavioural and physiological adaptation. Insects, due to their ecological importance, are especially affected by the widespread use of insecticides. While physiological resistance to insecticides is well documented, less is known about how such resistance influences behaviour, particularly oviposition site choice, a decision with direct consequences for offspring survival. Using *Drosophila melanogaster* , we investigated whether genetic resistance conferred by the detoxification gene *Cyp6g1* affects oviposition preferences and survival across life stages when exposed to insecticides. We presented resistant and susceptible female flies with a choice between food laced with acetone, insecticides to which they are resistant, or insecticides to which *Cyp6g1* does not confer resistance, and examined larval and adult survival under matching exposure conditions. We found that resistant females differ from susceptible flies by avoiding laying eggs on food containing DDT, an insecticide they are resistant to, suggesting that resistance is associated with a parallel shift in behaviour. Larval survival was closely tied to maternal oviposition choice, with *Cyp6g1*‐mediated resistance conferring survival benefits only against insecticides it can detoxify. In contrast, adult survival was less affected by genotype, highlighting the importance of oviposition site selection in shaping transgenerational fitness. Our results suggest that resistance alleles can impact not only physiological resistance but also incur behavioural adaptations such as toxin avoidance that act synergistically to mitigate insecticide exposure. Furthermore, our results show that these resistance alleles influence behaviour in ways that affect their frequency in natural populations.

## Graphical abstract

Using isogenic *Drosophila melanogaster* lines that differ at the well‐characterised Cyp6g1 resistance locus, we show that insecticide resistance is associated with altered oviposition site choice—an adaptive behaviour with transgenerational fitness consequences. Resistant females avoided laying eggs on substrates containing insecticides they can detoxify, thereby increasing offspring survival, but this phenomena does not happen in with their susceptible counterparts. These results highlight a novel behavioural dimension to resistance evolution and provide new insights into how maternal behaviour can amplify or mitigate the ecological costs of environmental toxins.

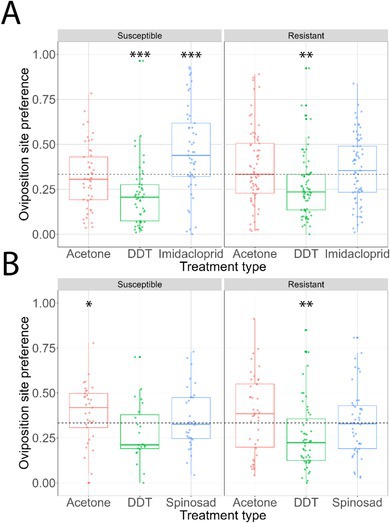

## 1 Introduction

Evolution shapes life on our planet by driving diversity and adaptation across ecosystems (Darwin 1959). However, these adaptations are constantly challenged by environmental pressures, even more so with the effect of anthropogenic action (Dawson et al. 2011; Foden et al. 2013; Thomas et al. 2004). One strategy through which animals can buffer the negative effects of anthropogenic action is through behavioural plasticity, an individual can change their behaviour depending on the environmental conditions it is subjected to (Mery and Burns 2010). Insects, which make up around 80% of all described species and are foundational to ecosystem health, are especially vulnerable to factors such as habitat loss (Wagner et al. 2021), increasing temperatures (Martelli et al. 2020; Van Heerwaarden and Sgrò 2021), nutritional stress (Chakraborty et al. 2023) and exposure to xenobiotic compounds (Sánchez‐Bayo and Wyckhuys 2021). For insects, the behaviours individuals exhibit under environmental pressures will therefore have critical impacts on the persistence and abundance of populations.

Oviposition behaviour, in particular, is a critical trait that interacts with environmental pressures. This key fertility trait impacts not only the environment females spend part of their lifespan but also determines the environment in which their progeny will develop (Nylin and Gotthard 1998; Silva‐Soares et al. 2017). Female insects often adjust their egg‐laying preferences to avoid unsuitable environments, increasing the likelihood of offspring survival (Nylin and Janz 1993; Wedell et al. 1997), or to colonise new environments where inter‐specific competition will be lowered (Silva‐Soares et al. 2017). This behaviour can be influenced by many factors, mostly linked to sensory stimuli. Olfactory cues such as linalool in hawkmoth‐pollinated flowers impact *Manduca sexta* oviposition preferences (Reisenman et al. 2010). On the other hand, several Lepidoptera (butterflies and moths) also use visual stimuli such as leaf shape and colour to make decisions about where to lay their eggs (Kelber 1999; Rausher 1978). Gustatory cues are some of the most important traits that influence oviposition behaviour, as these determine the food source the progeny will grow on, and insects have been known to choose food sources that maximise larval survival and optimise larval development (Rodrigues et al. 2015; Silva‐Soares et al. 2017). Gustatory cues can, however, be overridden if is associated with toxic compounds (de Carvalho and Mirth 2017).

Insecticides are some of the most toxic compounds for insects, and stand out as a major driver of insect population decline (Wagner et al. 2021). Amid this environmental crisis, evolution is finding ways to circumvent this issue, and some insect populations are evolving resistance to these xenobiotic compounds (Schmidt et al. 2010). An example of adaptation to the selective pressure that insecticides are is the *Cyp6g1* gene, a gene coding for a cytochrome P450 enzyme in *Drosophila melanogaster* that has been co‐opted as a detoxification mechanism (Schmidt et al. 2010). Variants of this gene confer resistance to multiple classes of insecticides, but not to more recently developed ones, as the flies have not been exposed sufficiently long to develop resistance to them (Battlay et al. 2016; Le Goff and Hilliou 2017; Schmidt et al. 2010). While the role of *Cyp6g1* in detoxification is well‐documented, less is known about how adaptation to insecticides through this gene will influence other traits, especially behavioural ones such as oviposition behaviour. Furthermore, if *Cyp6g1* confers any changes to behavioural traits, are these changes equally widespread to all insecticides, or just the ones it confers resistance to, and therefore context‐dependant?

Behavioural plasticity in oviposition choice has been extensively studied in relation to different environmental cues (Nylin and Gotthard 1998). However, little is known about how resistance to insecticides due to increased expression of *Cyp6g1* can influence oviposition behaviour and potentially mitigate some of the negative effects of exposure to toxins, complementing physiological resistance. Resistance to insecticides might incur changes in behaviour in one of two ways: (1) it might result in an increase in egg laying in environments insects are resistant to, which would result in less competition from susceptible conspecifics; (2) or it might result in avoidance of these food sources altogether, as they are recognised as toxic. Either of these scenarios will impact adult survival as fruit flies usually feed off the same sources as where they oviposit (Jaenike 1986). Additionally, larvae are restricted to feed off the substrate their parents chose to lay eggs in (Rodriguez et al. 1992), so choosing a food source with or without insecticides might have a major impact in larval survival. Understanding whether genes like *Cyp6g1* that confer insecticide resistance also influence adaptive behaviours such as oviposition could shed light on how insects survive in the presence of toxins.

This study investigates how genetic adaptations to insecticides, particularly through *Cyp6g1* expression, extend beyond detoxification to influence oviposition behaviour. Furthermore, we explore whether this gene confers changes in oviposition behaviour to all insecticides or only to the ones that *Cyp6g1* confers resistance to. This knowledge will provide insight into the broader ecological and evolutionary consequences of insecticide resistance and how resistant variants of this gene are spread. We use *D. melanogaster* to examine the oviposition preference for food laced with different insecticides that it is exposed to in natural populations. We exposed females to insecticides to which they have evolved resistance to; as well as novel insecticides to which they lack resistance. We further our investigation by also examine the resulting impact of exposure to these insecticides on larval and adult survival. We hypothesise that resistance to insecticides conferred by *Cyp6g1* may be coupled with changes in oviposition preferences and are dependent on the type of insecticide present. Any behavioural changes conferred to flies by a resistant *Cyp6g1* allele will be specific to insecticides for which *Cyp6g1* confers resistance to, and therefore other insecticides won't cause behavioural changes in oviposition. Furthermore, we hypothesise that these changes in behaviour will have further consequences for the survival of future generations. Larvae that are reared in environments with toxins to which *Cyp6g1* does not confer resistance to will have lower survival compared to larvae reared in environments with insecticides they are resistant to.

## 2 Methods

### 2.1 Fly Stocks

We used *D. melanogaster* isogenic lines available from the Australian Drosophila Isogenic Panel provided by Christen Mirth and Carla Sgrò (Monash University, Australia), and established according to Chakraborty et al. (2023). Flies were maintained at 25°C with a 12:12 h light: dark cycle on an SYA media containing 50 g of sugar, 100 g of yeast, 8 g of agar, 30 mL of Nipagin and 3 mL of propionic acid per litre of water.

### 2.2 Genotyping for *Cyp6g1* Alleles

To determine the *Cyp6g1* resistance allele of each isogenic line, we performed a single fly DNA extraction by squishing individual flies with 50 μL of a squish buffer containing 10 mM Tric‐Cl pH 8.2, 1 mM EDTA and 25 mM NaCl. Following this, 0.5 μL of Proteinase K was added and samples left to incubate at 37°C for at least 1 h. Once the incubation period was over, samples were subjected to 95°C for 15 min, followed by a centrifuging period of 3 min at 8000 rpm. The supernatant was then used for PCR diagnostic. Following DNA extraction, we performed a PCR with these samples using the primer sequences used in Schmidt et al. (2010). *Cyp6g1* alleles were annotated by comparing the gel images with the diagram present in Schmidt et al. (2010).

### 2.3 Oviposition Assays

We performed two types of oviposition choice assays: choice between insecticides that *Cyp6g1* confers resistance to, and choice between different resistance insecticides, including one that *Cyp6g1* does not confer resistance to. We treated the flies to three classes of widely used insecticides, ranging from organochlorines (DDT, an insecticide heavily used in the past, now banned in most countries, with exception of a few [Van den Berg 2009]), neonicotinoids (imidacloprid, one of the most widely used insecticides at the present [Ihara and Matsuda 2018]) and pediculicides (Spinosad, a recently discovered compound that has been increasing in usage worldwide [Hertlein et al. 2011]) to test where female adult flies prefer to lay their eggs. In *D. melanogaster* the *Cyp6g1* resistance alleles provide resistance to DDT and imidacloprid, but not to Spinosad (Battlay et al. 2016; Battlay et al. 2018; Daborn et al. 2002; Daborn et al. 2007; Le Goff et al. 2003; Le Goff and Hilliou 2017). Several genotypes were tested ranging from insecticide susceptible flies (*Cyp6g1*—M allele), and insecticide resistant flies (*Cyp6g1*—AA, BA and BP alleles). We selected 10 lines from the isogenic panel, with each line carrying one of the four *Cyp6g1* alleles present (3 homozygous M lines, 3 homozygous AA lines, 2 homozygous BA lines and 2 homozygous BP lines), and reared them in density‐controlled vials in the conditions described above. After eclosion, flies from each isogenic line were transferred to separate fresh vials and aged to 4–5 days old where they could mate *ad libitum*.

Once the ageing period was completed, we transferred 20 female and 5 male flies from each isogenic line to separate oviposition arenas with three Eppendorf caps containing food laced with different compounds, glued to a petri dish (6 cm diameter), capped by a 200 mL plastic cup. Males were included in this assay to mimic environmental conditions and allow females unrestricted mating to stimulate oviposition. All caps contained the same media, made with the same recipe as mentioned above. For the first assay involving insecticides *Cyp6g1* alleles confer resistance to, the lids contained 5 μL of one of three compounds: acetone (the control group), DDT dissolved in acetone, or imidacloprid dissolved in acetone. Both DDT and imidacloprid were dissolved to achieve a final concentration of 1 ppm/mL of media, concentrations known to be found in nature (Boul et al. 1994; Knoepp et al. 2012). The solutions were put on top of the food 1 h before the start of the assay to allow all acetone to evaporate. For the second assay, involving Spinosad, an insecticide that *Cyp6g1* does not provide resistance to, loose microtube caps were used, containing 350 μL of SYA media and 5 μL of one of three compounds: acetone (control group), DDT dissolved in acetone, or Spinosad dissolved in acetone. The final concentrations achieved for these compounds were 1 ppm for DDT and 0.5 ppm for Spinosad, concentrations known to be found in nature (Sharma et al. 2007). Despite different volumes of food between experiments, the surface area exposed to flies remained the same. Solutions were put on 1 h before the start of the assay to allow all acetone to evaporate. Flies were allowed to interact and lay eggs for 24 h, after which flies were discarded, and eggs in each cap counted.

### 2.4 Larval and Adult Toxicology Assays

To measure larval mortality, we allowed flies from each isogenic line to lay eggs on an apple juice agar plate containing 12.5 g of agar, 150 mL apple juice (Cole's brand, Australia), 275 mL of water and 10.5 mL of Nipagin. After 24 h, 30 L1 stage larvae were picked and put in vials containing 5 mL of SYA media to which 5 μL of either acetone, DDT, imidacloprid, or Spinosad were rolled onto the vial and food to reach the concentrations described above. Larvae were allowed to feed and develop to adulthood. After 10 and 15 days, the number of eclosed adult flies was counted.

To measure male and female mortality, we allowed flies from each isogenic line to lay eggs in vials containing SYA media. Once flies eclosed, 20 female and 5 male flies were housed together to mimic the conditions set for the oviposition assays in a vial containing 3 mL of SYA media and laced with 3 μL of one of four compounds: acetone, DDT, imidacloprid, or Spinosad to the same final concentrations as described above. The number of surviving flies from each sex was counted after 7 days.

All vials with SYA media were laced with the respective insecticide or acetone at least 1 h before the start of the assays to allow for all acetone to evaporate. All assays were performed at 25°C with a 12:12 h light: dark cycle.

### 2.5 Statistical Analysis

To test for differences in oviposition preference, we first filtered the data excluding any samples that had less than an average of 1 egg laid per female, since a preference outcome would show at least 1 egg laid per female in any food (50 out of 118 samples excluded from the imidacloprid preference assay, 46 out of 119 samples excluded from the Spinosad preference assay). To first test for differences between the two assays, we fit a generalised linear mixed model (lme4 package, Bates et al. 2015) with the response variable being the proportion of eggs laid in each treatment. Experiment (imidacloprid or Spinosad experiment), chemical compound and susceptibility status (either susceptible or resistant) were considered fixed effects, with corresponding interaction terms considered, and each isogenic line, nested within the corresponding allele, and in turn, nested within each corresponding susceptibility status were fitted as random effects together with replicate.

To test differences within each experiment, we fit a generalised linear mixed model with an integrated template model builder (glmmTMB package, Brooks et al. 2017), with the response variable being the proportion of eggs laid in each treatment. Chemical compound and susceptibility status (either susceptible or resistant) were considered fixed effects, and each isogenic line, nested within the corresponding allele, and in turn nested within each corresponding susceptibility status was fitted as random effect together with replicate. Due to overdispersion of the data, a beta‐binomial distribution was assumed. Post hoc comparisons of the means to 0.33 (meaning no preference), and corresponding odds ratios were conducted using the emmeans function (lsmeans package, Lenth 2016).

For larval, female and male adult survival, we fit a generalised linear mixed model with an integrated template model builder (glmmTMB package), with the response variable being the proportion of live individuals in each replicate at the end of the assay. Chemical compound and susceptibility status were considered as fixed effects, and each isogenic line, nested within the corresponding allele was fitted as random effect, alongside with replicate. Post hoc comparisons between treatments, and corresponding odds ratios were conducted using the emmeans function (emmeans package).

In all analyses, the allele factor was taken into account as a random effect to remove any differences observed between resistant alleles, since any of those alleles would confer resistance to DDT and imidacloprid, and differences between alleles were outside the scope of this study.

Data was analysed and visualised in R Studio (version 3.4.1). Plots were produced using ggplot2 (tidyverse package, Wickham 2011). All data and R scripts are available on Figshare (https://doi.org/10.26188/29848040.v1).

## 3 Results

### 3.1 Presence of Resistance Allele Changes Oviposition Preference

We hypothesised that the presence of a *Cyp6g1* resistance allele in *D. melanogaster* would change oviposition preference, and that this change would be dependent on whether or not the resistance allele provided resistance to the toxin. To test this prediction, we allowed susceptible and resistant females to lay eggs indiscriminately in media containing no insecticides, insecticides that resistant flies are resistant to and insecticides that *Cyp6g1* does not confer resistance to.

We detected a significant effect from the three‐way interaction between susceptibility, compound and experimental setup, indicating that this behaviour is context‐dependant, and also depends on the flies' susceptibility status and the combination of compounds they have access to (Table S1). This result indicates some measure of behavioural plasticity, and that susceptible flies change their behaviours depending on the options offered, whereas resistant females do not change their preference on where to oviposit (Table S1). To further investigate these interactions, we performed further analysis on each experiment separately.

**FIGURE 1:**
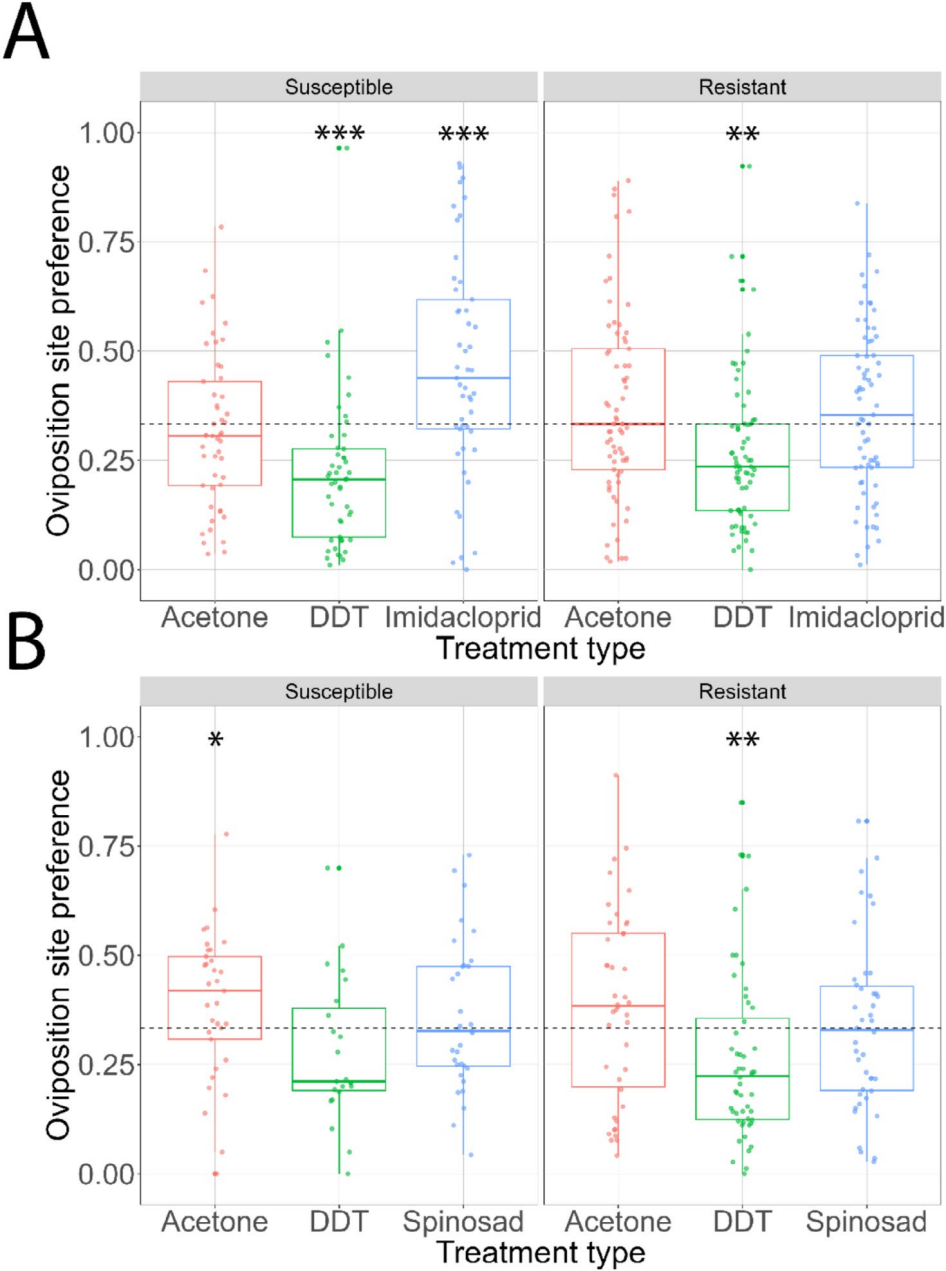
Oviposition preference between foods laced with no insecticides or different classes of insecticides. Susceptible and resistant flies were given three choices on where to lay eggs. (A) Food laced with acetone (red boxplot), the solvent control, food laced with DDT (green boxplot) and food laced with imidacloprid (blue boxplot), both insecticides that are known to be metabolised by *Cyp6g1*. (B) Food laced with acetone (red boxplot), the solvent control, food laced with DDT (green boxplot), an insecticide known to be metabolised by *Cyp6g1* and food laced with Spinosad (blue boxplot), an insecticide that *Cyp6g1* does not metabolise. Dashed line represents 33% which signifies no preference to lay eggs in one site over another. ***p‐value < 0.001; **p‐value < 0.01; *0.05 < *p*‐value < 0.01.

We observed that the proportion of eggs laid significantly changes depending on the choices offered for both assays (Table 1). Furthermore, the presence of a resistance allele in these flies alters their response to the treatments, resulting in a significant compound by susceptibility interaction when exposed to imidacloprid (Table 1). We also found that when faced with a choice between media containing no insecticides, media with DDT, or with imidacloprid, two insecticides that *Cyp6g1* resistance alleles confer resistance to, susceptible flies are 41% less likely to choose food sources laced with DDT but have a 69% chance of preferring to lay eggs in food sources laced with imidacloprid (Figure 1, Table 1). Resistant flies, however, only actively avoid DDT‐laced foods, and are 22% less likely to lay eggs in DDT‐laced food (Figure 1, Table 1).

**TABLE 1:** Effect of chemical compound and fly insecticide susceptibility on oviposition preference for food laced with acetone, the solvent control, food laced with DDT or imidacloprid, both insecticides that are known to be metabolised by *Cyp6g1*, and food laced with Spinosad, an insecticide that *Cyp6g1* does not metabolise. Least‐square mean comparison between treatments and no preference (33%) and corresponding odds ratio.

Acetone vs. DDT vs. imidacloprid			
Proportion of eggs ~ compound × susceptibility + (1|susceptibility/allele/line) + (1|replicate)			
	Chi‐squared	df	*p*
Intercept	35.1920	1	< 0.0001***
Compound	34.7527	2	< 0.0001***
Susceptibility	1.5476	1	0.2135
Compound × susceptibility	10.6701	2	0.0048**

Susceptibility								
Compound	Susceptible				Resistant			
	Lsmean	St error	*p*	Odds ratio against 0.33	Lsmean	St error	*p*	Odds ratio against 0.33
Acetone	−0.729	0.123	0.8640	0.966	−0.530	0.103	0.0842	1.178
DDT	−1.267	0.126	< 0.0001***	0.594	−0.972	0.102	0.0094**	0.784
Imidacloprid	−0.169	0.127	< 0.0001***	1.685	−0.551	0.110	0.1547	1.129

Acetone vs. DDT vs. spinosad			
Proportion of eggs ~ compound × susceptibility + (1|susceptibility/allele/line) + (1|replicate)			
	Chi‐squared	df	*p*
Intercept	10.0838	1	0.0015**
Compound	6.3120	2	0.0425*
Susceptibility	0.0496	1	0.8238
Compound × susceptibility	0.1517	2	0.9270

Susceptibility								
Compound	Susceptible				Resistant			
	Lsmean	St error	*p*	Odds ratio against 0.33	Lsmean	St error	*p*	Odds ratio against 0.33
Acetone	−0.430	0.140	0.0471*	1.298	−0.470	0.128	0.0625	1.246
DDT	−0.972	0.178	0.1371	0.753	−1.031	0.123	0.0089**	0.710
Spinosad	−0.595	0.132	0.2779	1.146	−0.705	0.123	0.9787	0.999

* 0.05 < *p*‐value < 0.01.

** *p*‐value < 0.01.

*** *p*‐value < 0.001.

When faced with a choice between food sources laced with either no insecticides, an insecticide that resistant flies are resistant to, and an insecticide that *Cyp6g1* does not confer resistance to, we observed that the proportion of eggs laid significantly changed depending on the chemical compound the food was laced with. The preference of these flies to lay eggs, although not statistically significant when comparing insecticide susceptibility, seems to differ depending on the compound offered (Table 1). Susceptible flies prefer to lay eggs in acetone‐laced food, with a 30% chance of increased egg laying in this food type and have no preference for or avoidance of DDT‐laced food. While resistant flies avoid laying eggs in DDT‐laced food, with a 29% chance of laying less eggs in foods laced with this insecticide they do not prefer acetone‐laced food. Additionally, both types of flies show no oviposition preference for Spinosad‐laced food (Figure 1, Table 1). These results indicate that resistant flies do not alter their preference of avoiding DDT‐laced food, whereas susceptible flies shift their preference from avoiding DDT‐ and preferring imidacloprid‐laced food, to only preferring acetone‐laced food in the presence of Spinosad.

### 3.2 Survival at Different Life Stages Is Resistance‐Dependent

To characterise differences in larval and adult survival for flies differing in their susceptibility status, we submitted freshly hatched larvae and freshly eclosed adults to food sources containing only one of the compounds used in the oviposition preference assays and recorded survival.

**FIGURE 2:**
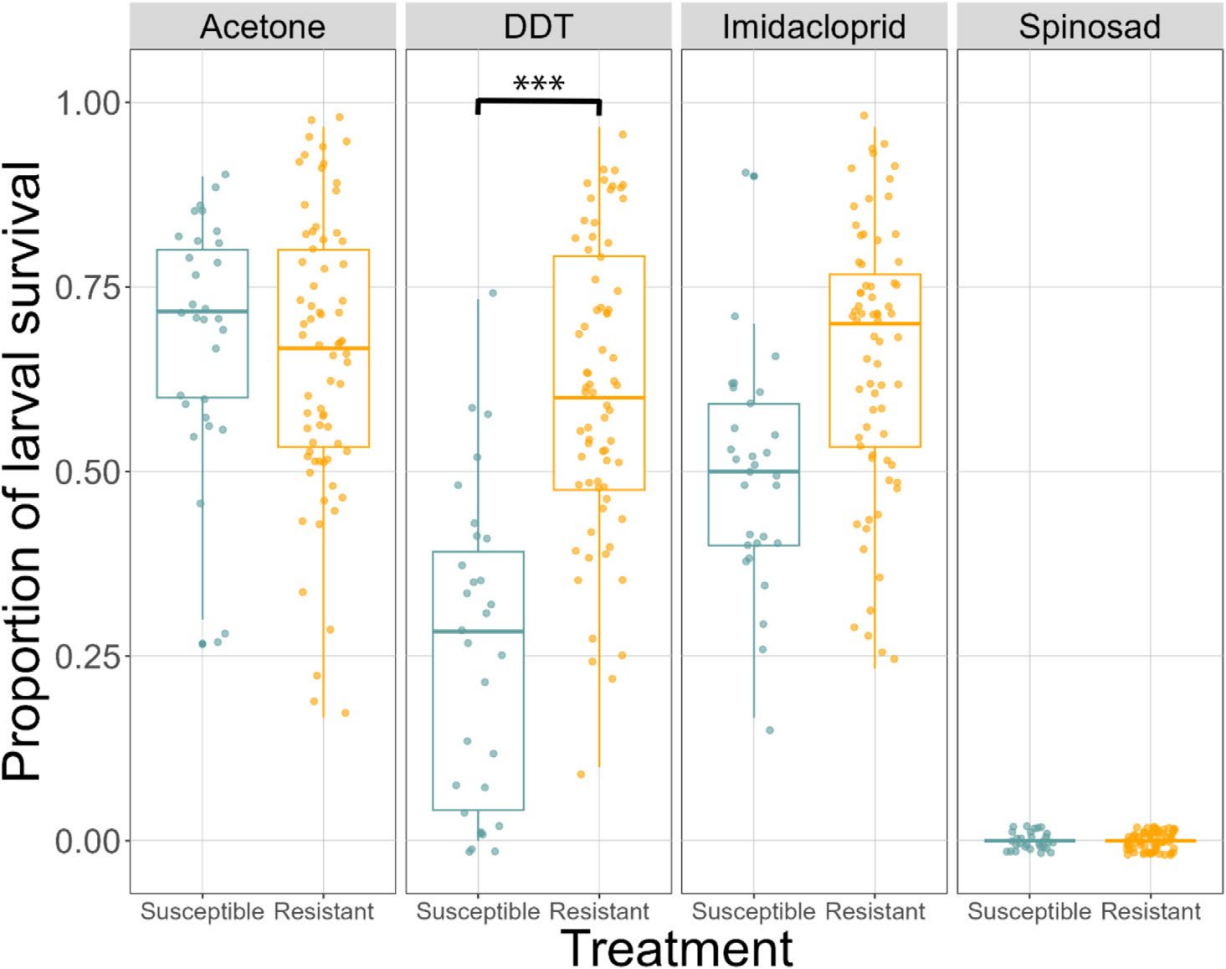
Larval survival in food laced with different toxic compounds. Freshly hatched susceptible (blue) and resistant (orange) larvae were reared in media containing one of four compounds: Acetone (Control), DDT, Imidacloprid and Spinosad. ***p‐value < 0.001; **p‐value < 0.01; *0.05 < *p*‐value < 0.01.

Susceptible and resistant larvae have similar survival rates across treatments, as shown by a non‐significant effect of susceptibility status (Table S2). The different compounds impact larval survival differently, with our control treatment, food laced with acetone having the highest survival, followed by DDT, imidacloprid and Spinosad having the least number of larvae surviving to adulthood (Figure 2, Table S2). There is a significant susceptibility status by chemical compound interaction, which is exemplified by the DDT‐laced food, where, on average, only 25% of the susceptible larvae survived, compared to roughly 60% for resistant larvae (Figure 2, Table S2). Both acetone‐laced food and imidacloprid‐laced food incurred in similar survival rates for both susceptible and resistant larvae. On these food sources, survival rates range from 50%–79% (Figure 2). Only Spinosad‐ laced food caused 100% mortality in both types of larvae (Figure 2).

When recording adult female survival, we observed a significant effect of compound‐laced foods, which is shown by different survival rates across the different compound‐laced foods (Figure S1, Table S3). When investigating further the effect of each compound, we observed that acetone‐ and DDT‐laced food caused no difference in survival between susceptible and resistant flies (Figure S1, Table S3). The survival across treatments ranged between 75 to 100% for both susceptible and resistant female flies. However, imidacloprid‐ and Spinosad‐laced food significantly impacted susceptible female flies more severely, causing higher mortalities, with Spinosad‐laced food causing roughly 80% mortality in susceptible female flies, compared with a mean mortality of roughly 25% in resistant flies (Figure S1, Table S3).

For male adult survival, we only observed a significant effect of the compound the foods were laced with (Table S4), whereas the insecticide susceptibility status, and the interaction between susceptibility status and chemical compound were non‐significant. This suggests that susceptible and resistant male adults respond to the compounds similarly (Figure S2, Table S4). When observing odds ratio of each treatment we conclude that acetone‐ and DDT‐laced foods do not impact susceptible and resistant flies differently (Figure S2, Table S4). There is, however, a higher variability in male survival compared to females across all compounds (Figure S2). Additionally, and similar to female adults, imidacloprid‐ and Spinosad‐laced foods cause a higher mortality of susceptible males, with Spinosad‐laced food causing close to 100% mortality (Figure S2, Table S4).

## 4 Discussion

Evolutionary responses to environmental pressures, especially those of anthropogenic origin, can drive rapid physiological and behavioural changes in affected populations (Wong and Candolin 2015). Insecticide deployment is a powerful selective force, shaping resistance evolution across many insect species (Feyereisen 1995). The *Cyp6g1* gene in *D. melanogaster* is one of the best‐documented examples of such adaptive evolution (Schmidt et al. 2010). In this study, we investigated how insecticide resistance at the *Cyp6g1* locus affects oviposition behaviour, larval survival and adult survival in the presence of insecticides. Importantly, we used concentrations of DDT, imidacloprid and Spinosad comparable to those found on crops after application, simulating realistic environmental exposure (Boul et al. 1994; Knoepp et al. 2012; Sharma et al. 2007). Because oviposition site selection directly affects offspring traits such as survival, development and body size (Nylin and Gotthard 1998; Rodrigues et al. 2015; Silva‐Soares et al. 2017), behavioural variation in oviposition choice can significantly influence fitness. Toxin avoidance, or not discriminating between toxins when resistant to them, can represent a major adaptation potentially impacting the frequency of resistance alleles in natural populations.

### 4.1 Insecticide Resistance Alters Oviposition Preference

Previous research has found that insecticide resistance provided by the *Cyp6g1* locus is capable of altering behaviours in *D. melanogaster* such as male courtship (Rostant et al. 2017). In this study we found further evidence that the presence of a resistance allele strongly influences another behavioural trait, oviposition preference. Resistant females consistently avoided food laced with DDT, an insecticide to which they are resistant. This suggests that resistance is not simply a matter of detoxification but may also involve evolved behavioural adaptations such as insecticide avoidance. Furthermore, the choices made by female flies on where to oviposit eggs have greater impacts not only on their own survival but also dictate the survival of their progeny.

Oviposition behaviour is known to be plastic and adaptive, changing in response to environmental cues such as nutrient availability, microbial presence, chemical deterrents and volatile cues (Akhtar and Isman 2003; Fowler et al. 2025; Silva‐Soares et al. 2017; Tungadi et al. 2023). In our experiment, acetone‐treated food served as a control. When given a choice between acetone, DDT and imidacloprid laced foods, both genotypes had no preference for acetone‐laced food but significantly avoided DDT‐laced food. In addition, susceptible flies also showed a preference for imidacloprid‐laced food. Imidacloprid is classified as a neonicotinoid and acts in a similar fashion to nicotine and has been found to have addictive effects in insects (Kessler et al. 2015). This could potentially explain why susceptible flies, which cannot metabolise imidacloprid, have a marked preference to lay eggs in that food source. Resistant flies, in contrast, have a higher *Cyp6g1* expression which allows for the detoxification of this compound in the gut and malpighian tubules before it reaches the brain and impacts behaviours (Fusetto et al. 2017; Schmidt et al. 2010).

In contrast, when given a choice between an insecticide that *Cyp6g1* can metabolise (DDT) and one it cannot (Spinosad), resistant genotypes again avoided laying eggs on food with the insecticide they were resistant to and had no preference for the non‐metabolizable insecticide, reinforcing the idea that evolved resistance associated with *Cyp6g1* is coupled with risk‐avoidant behavioural shifts. This finding indicates a level of discriminatory oviposition behaviour that may be adaptive, by minimising offspring exposure to more harmful toxins. Perhaps resistant females also harbour a cost for detoxifying DDT, which might not be directly linked to survival, but with underlying phenotypic traits such as brain and gut function or immunity (Martelli et al. 2020), which were not observed in this study. And, as such, by avoiding toxins altogether, that cost is eliminated.

A change in behaviour that is context‐dependant has been observed previously in bees, called the decoy effect (Hemingway et al. 2024). This effect states that an organism's preference changes between higher‐value options when a lower‐value option (decoy) gets introduced. In the case of our study, adding a food laced with a compound *Cyp6g1* does not confer resistance to, alters the behaviour of susceptible flies who start preferring to lay eggs in a non‐toxic food, but no longer avoid the previously toxic food (DDT). The Spinosad‐laced food may act as a decoy, shifting preferences of susceptible flies who start preferring to lay eggs in a non‐toxic food, but no longer avoid the previously toxic food (Figure 1, Table 1, Hemingway et al. 2024). This indicates that Genotype × Environment interactions in the form of behavioural plasticity exist. The behaviour of *D. melanogaster* flies is context dependant, which has been observed before in the context of social experience (Chen and Sokolowski 2022). Courtship behaviour in *D. melanogaster* males changes if a male is courting an unmated female vs. a mated one (Chen and Sokolowski 2022). Similarly, adult lifespan is also impacted by the presence or absence of conspecifics for these fruit‐flies (Chen and Sokolowski 2022). However, oviposition behaviour had never before been documented in the context of insecticide resistance.

### 4.2 Behavioural Adaptations Carry Consequences for Larval and Adult Life Stages

Larval development is entirely tied to the oviposition site chosen by the mother in many insects, meaning the maternal decision has long‐lasting consequences for offspring (Paukku and Kotiaho 2008). Our results showed that larval survival varied significantly across treatments and genotypes. Resistance alleles at *Cyp6g1* conferred a clear fitness advantage when larvae were reared on food containing DDT or imidacloprid but offered no protection against Spinosad—reinforcing that *Cyp6g1* does not confer resistance to this insecticide (Daborn et al. 2007; Le Goff et al. 2003; Le Goff and Hilliou 2017). This indicates that existing resistance alleles provide no cross‐protection to more recently developed compounds, reflecting the continual evolutionary arms race between insects and chemical control strategies. This places strong selection on populations to evolve novel resistance mechanisms (Ffrench‐Constant 2007; Mallet 1989).

Susceptible larvae that are reared in substrates laced with toxins have lower survival rates compared with resistant larvae. Despite observing no cost in terms of survival for resistant larvae when reared on foods containing insecticides, *Cyp6g1* confers resistance to in our study; this does not mean there are no associated costs for these flies. Previous studies have shown that there are adult carry‐on costs of larvae exposed to insecticides such as imidacloprid, and these phenotypic costs range from locomotion impairments, lower success in mating, as well as courtship behaviour impairments (Young et al. 2020). This indicates that an adult reared in insecticide‐laced food will perform worse than one reared in an insecticide‐free food in many traits directly linked to fitness, even when they are resistant to these insecticides. Ultimately, avoiding insecticides altogether might present as the best strategy for survival and lifetime fitness.

Unlike larvae, adult *Drosophila* are mobile and can escape or avoid contaminated environments. In our study, we restricted flies to one environment, and we found that adult survival, unlike larval survival, was not significantly influenced by resistance genotype when exposed to residual levels of DDT. Both sexes survived similarly across treatments, suggesting that at these doses, DDT is not sufficiently toxic to elicit genotype‐specific mortality effects. Imidacloprid and Spinosad were more potent. Interestingly, although *Cyp6g1* does not confer known resistance to Spinosad, we observed a modest survival benefit in adult flies carrying resistance alleles. This may suggest low‐level recruitment of detoxification pathways, potentially providing slight delays in toxicity effects—a phenomenon seen in other studies of pleiotropic effects of xenobiotic resistance alleles (Coustau and Chevillon 2000; Dyer 2018). Research has shown that the environment in which adults choose to lay eggs is known to have transgenerational consequences (Fox et al. 1995). In seed beetles, not only the species of plant, but also if that hostplant is exposed or not impacts progeny survival and predation, which ultimately will impact a population's persistence in the environment (Fox et al. 1995; Tschanz et al. 2005).

Our results reinforce the idea that life‐stage ecology (i.e., sedentary larvae vs. mobile adults) plays a major role in shaping the evolutionary impact of toxic exposure, and that resistance‐associated behaviours such as oviposition preference may have greater fitness consequences than adult physiological resistance per se (Sparks et al. 1989; Zalucki and Furlong 2017). Resistant females will avoid food sources that are toxic, even if they are resistant to the compound, and in turn this choice will consequently increase their progenies' survival. Susceptible flies on the other hand, do not make the same discrimination. Because of this lack of avoidance, the progeny that develop in toxic environments have lower survival rates, reducing the frequency of susceptible *Cypg61* alleles in natural populations. The presence of a *Cyp6g1* resistance allele creates a shift in oviposition behaviour, towards avoiding toxic environments that will have a drastic impact on larval survival.

Our study shows that evolutionarily derived resistance at the *Cyp6g1* locus shapes behavioural plasticity for oviposition preference, leading resistant females to avoid food that is laced with insecticides they are resistant to. This allows for a higher chance of survival, since there is less contact with the toxic compound, minimising the cost of detoxification and also increasing survival for their progeny. This behavioural adaptation has direct implications for offspring fitness, particularly since insecticides persist in the substrate. Changes in oviposition preference that impact life‐history traits of offspring, and ultimately their fitness, have been observed in different classes of insects (Gibbs et al. 2018; Harris et al. 2001; Nylin and Janz 1993; Reguera and Gomendio 2002). This study shows how insecticides ultimately present themselves as another compound in plants to which insects will have to adapt for their species to persist. Our research further highlights the importance of oviposition preference as an adaptive behaviour with trans‐generational impacts. These findings highlight the intersection between insecticide resistant alleles and the ecological and evolutionary importance of oviposition site choice in mediating the effects of environmental toxins, especially when future generations will be constrained to the maternal decision. Considering resistance to insecticides keeps increasing and expanding to new species (Bass et al. 2015), comprehending how insects will adapt to the presence of these compounds is paramount to understand the persistence of populations.

Our findings emphasise that understanding insect adaptation to anthropogenic action through xenobiotics requires a holistic approach—incorporating behavioural ecology, physiological resistance and life‐history dynamics. As agricultural practices continue to apply selective pressure on insect populations, it is crucial to consider how evolutionary adaptations unfold not just at the molecular level, but across the full life‐history and generational scale. By integrating behaviour, survival and genetic resistance, our study contributes to a broader understanding of how insects adapt to chemical stressors and highlights the complex interplay between evolution, ecology and life‐stage‐specific selection.

## Author Contributions

**A. Nogueira Alves:** conceptualization (lead), data curation (lead), formal analysis (lead), investigation (lead), methodology (lead), visualization (lead), writing – original draft (lead), writing – review and editing (lead). **N. Wedell:** conceptualization (equal), funding acquisition (lead), methodology (equal), project administration (lead), supervision (lead), writing – original draft (equal), writing – review and editing (lead). **F. Martelli:** conceptualization (equal), data curation (equal), investigation (equal), methodology (equal). **Y. T. Yang:** data curation (equal), investigation (equal), methodology (equal).

## Conflicts of Interest

The authors declare no conflicts of interest.

## Supporting information


**Appendix S1:** ece373067‐sup‐0001‐AppendixS1.docx.

## Data Availability

All data and R scripts are available on Figshare (https://doi.org/10.26188/29848040.v1).
