# Supplementary material for: Insecticide Resistance Alters Oviposition Preference in Drosophila melanogaster
Source: Ecol Evol. 2026 Feb 9;16(2):e73067. doi: 10.1002/ece3.73067 (PMC12884133; doi:10.1002/ece3.73067)
Supplement: Supplementary file 1 — Appendix S1: ece373067‐sup‐0001‐AppendixS1.docx. [file ECE3-16-e73067-s001.docx]

**Supplementary Material**

Supplementary Table 1: Effect of experimental context on oviposition behaviour. Experimental setup, chemical compound, and susceptibility status were tested for their effect on oviposition behaviour.

| Experiment-dependant context | | | |
| --- | --- | --- | --- |
| Proportion of eggs ~ Experiment * Compound * Susceptibility + (1\|Susceptibility/Allele/Line) + (1\|Replicate) | | | |
|  | Chi-squared | Df | P-value |
| Experiment | 133.5755 | 1 | <0.0001 *** |
| Compound | 573.3945 | 2 | <0.0001 *** |
| Susceptibility | 0.3169 | 1 | 0.5735 |
| Experiment * Compound | 25.1567 | 1 | <0.0001 *** |
| Experiment * Susceptibility | 65.6341 | 1 | <0.0001 *** |
| Compound * Susceptibility | 23.1688 | 2 | 0.0001 *** |
| Experiment * Compound * Susceptibility | 10.1527 | 1 | <0.0014 ** |

Supplementary Table 2: Effect of resistance status and treatment on larval survival and differences between resistant and susceptible larvae to different chemical compounds.

| Larval Survival | | | |
| --- | --- | --- | --- |
|  | Chi-squared | Df | P-value |
| Intercept | 4.0705 | 1 | 0.0436 * |
| Compound | 137.6867 | 3 | <0.0001 *** |
| Susceptibility | 0.0384 | 1 | 0.8446 |
| Compound * Susceptibility | 81.6971 | 3 | <0.0001 *** |
| Susceptible-Resistant | | | |
|  | Odds Ratio | SE | P-value |
| Acetone | 1.097 | 0.5180 | 0.8446 |
| DDT | 0.175 | 0.0830 | 0.0002 *** |
| Imidacloprid | 0.497 | 0.234 | 0.1374 |
| Spinosad | 6.622 | 0.00 | 0.9990 |

Supplementary Table 3: Effect of status and treatment on female adult survival and differences between resistant and susceptible adult females to different chemical compounds.

| Female adult survival | | | |
| --- | --- | --- | --- |
|  | Chi-squared | Df | P-value |
| Intercept | 20.8936 | 1 | <0.0001 *** |
| Compound | 211.5541 | 3 | <0.0001 *** |
| Susceptibility | 1.5633 | 1 | 0.2112 |
| Compound * Susceptibility | 5.6018 | 3 | 0.1327 |
| Susceptible-Resistant | | | |
|  | Odds Ratio | SE | P-value |
| Acetone | 0.421 | 0.291 | 0.2112 |
| DDT | 0.270 | 0.184 | 0.0549 |
| Imidacloprid | 0.153 | 0.103 | 0.0055 ** |
| Spinosad | 0.195 | 0.123 | 0.0098 ** |

Supplementary Table 4: Effect of status and treatment on male adult survival and differences between resistant and susceptible adult males to different chemical compounds.

| Male adult survival | | | |
| --- | --- | --- | --- |
|  | Chi-squared | Df | P-value |
| Intercept | 13.6857 | 1 | 0.0002 *** |
| Compound | 59.5288 | 3 | <0.0001 *** |
| Susceptibility | 0.4493 | 1 | 0.4493 |
| Compound * Susceptibility | 6.9431 | 3 | 0.0737 |
| Susceptible-Resistant | | | |
|  | Odds Ratio | SE | P-value |
| Acetone | 0.5514 | 0.4339 | 0.4493 |
| DDT | 0.2544 | 0.1793 | 0.0521 |
| Imidacloprid | 0.1443 | 0.1053 | 0.0080 ** |
| Spinosad | 0.0738 | 0.0627 | 0.0022 ** |


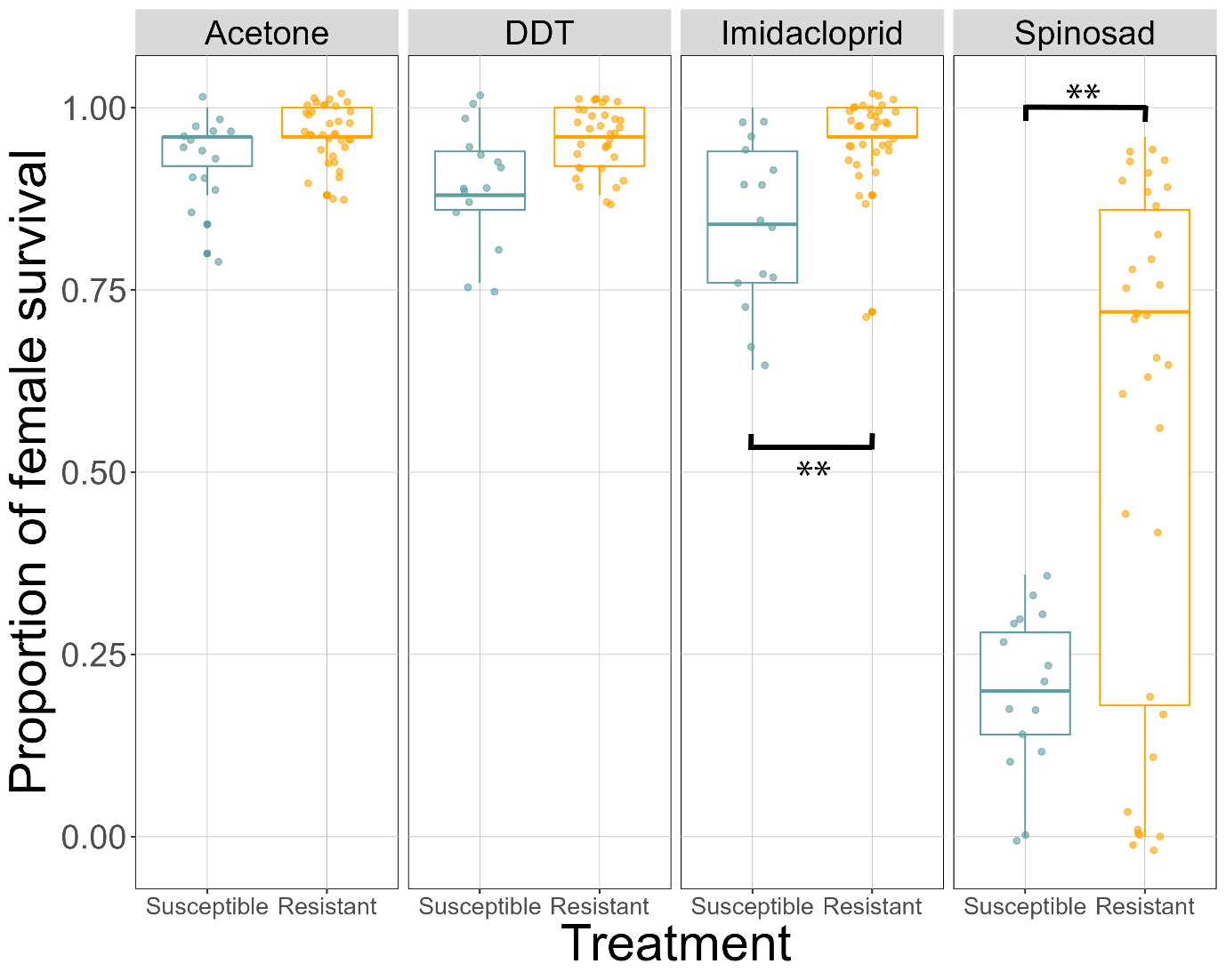


Supplementary Figure 1: Adult female survival in food laced with different compounds. Susceptible (blue), and resistant (orange) female flies were subjected to media containing one of four compounds: Acetone (Control), DDT, Imidacloprid, and Spinosad. *** - p-value<0.001; **p-value<0.01; * - 0.05<p-value<0.01


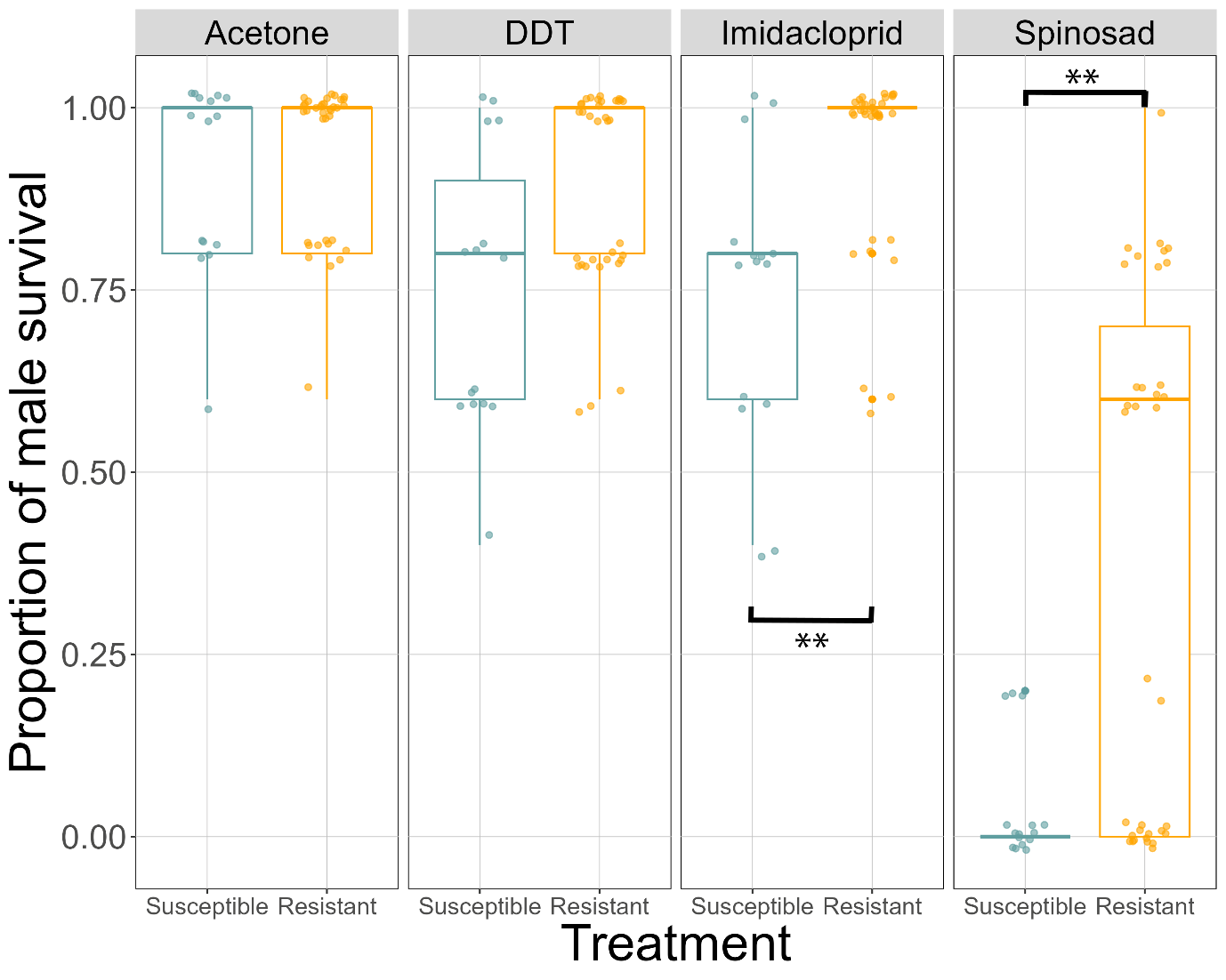


Supplementary Figure 2: Adult male survival in food laced with different compounds. Susceptible (blue), and resistant (orange) male flies were subjected to media containing one of four compounds: Acetone (Control), DDT, Imidacloprid, and Spinosad. *** - p-value<0.001; **p-value<0.01; * - 0.05<p-value<0.01
